# Mesenchymal Stem Cell Therapy: Hope for Patients With Systemic Lupus Erythematosus

**DOI:** 10.3389/fimmu.2021.728190

**Published:** 2021-09-30

**Authors:** Aifen Li, Fengbiao Guo, Quanren Pan, Shuxian Chen, Jiaxuan Chen, Hua-feng Liu, Qingjun Pan

**Affiliations:** Key Laboratory of Prevention and Management of Chronic Kidney Disease of Zhanjiang City, Institute of Nephrology, Affiliated Hospital of Guangdong Medical University, Zhanjiang, China

**Keywords:** systemic lupus erythematosus, mesenchymal stem cells, immunomodulation, transplantation, inefficacy, modification

## Abstract

Systemic lupus erythematosus (SLE) is a chronic autoimmune disease. Although previous studies have demonstrated that SLE is related to the imbalance of cells in the immune system, including B cells, T cells, and dendritic cells, etc., the mechanisms underlying SLE pathogenesis remain unclear. Therefore, effective and low side-effect therapies for SLE are lacking. Recently, mesenchymal stem cell (MSC) therapy for autoimmune diseases, particularly SLE, has gained increasing attention. This therapy can improve the signs and symptoms of refractory SLE by promoting the proliferation of Th2 and Treg cells and inhibiting the activity of Th1, Th17, and B cells, etc. However, MSC therapy is also reported ineffective in some patients with SLE, which may be related to MSC- or patient-derived factors. Therefore, the therapeutic effects of MSCs should be further confirmed. This review summarizes the status of MSC therapy in refractory SLE treatment and potential reasons for the ineffectiveness of MSC therapy from three perspectives. We propose various MSC modification methods that may be beneficial in enhancing the immunosuppression of MSCs in SLE. However, their safety and protective effects in patients with SLE still need to be confirmed by further experimental and clinical evidence.

## SLE Treatment Has a Long Way to Go

Systemic lupus erythematosus (SLE) is an autoimmune disease that exhibits high population heterogeneity, with women of childbearing age being the most highly affected population. The pathogenesis of SLE remains unclear. Previous studies showed that abnormal activation of immune cells, such as B cells (1), T cells (2), macrophages (3), basophils (4) and dendritic cells (DCs) (5), etc., played a crucial role in SLE. These activated immune cells also contributed to the production of pro-inflammatory factors and pathogenic autoantibodies, causing the deposition of immune complexes in tissues and inducing multiple organ damage. SLE is difficult to diagnose in the early stages because the symptoms and signs are not typical. Currently, the classic methods for SLE treatment are corticosteroids and immunosuppressors, which chronically prolong the disease course and mostly exhibit chronic remission-relapse, whereas a few patients achieve long-term remission (6). Importantly, immunosuppressive therapies fail to prevent disease relapse in more than half of the patients, and high-dose treatment may even increase the risk of severe infection and death (7). Additionally, most patients exhibit damage to the kidneys or other organs, partly limiting the application of immunosuppressive therapy. Hence, the development of new drugs and therapies is urgently needed (8) and especially the biological agents have gained the attention of researchers. In the past 60 years, belimumab has been the only biological agent approved by the US FDA for SLE treatment; however, this agent utilizes a single target and cannot inhibit plasma cells and switched memory B cells (9). Also, other biological agents, such as tabalumab, do not significantly improve the disease conditions and even have adverse side effects in patients with SLE (10).

Lymphopenia or leukopenia has been reported in patients with autoimmune diseases, such as SLE (11). Therefore, autologous hematopoietic stem cell (HSC) transplantation performed for SLE treatment (12). However, it was demonstrated that this therapy had high transplant-related mortality and relapse (13), possibly because of defects in the bone marrow stem cells and abnormal immune function in patients with SLE (14). Later, it was revealed that both genetic and inflammatory factors altered the number and function of HSCs in a murine lupus model (15). Also, it has been reported that allogeneic HSC transplantation caused relapses and opportunistic infections after seven months, which did not significantly differ from the adverse effects of autologous transplantation (16). Therefore, SLE treatment remains challenging.

**Figure 1:**
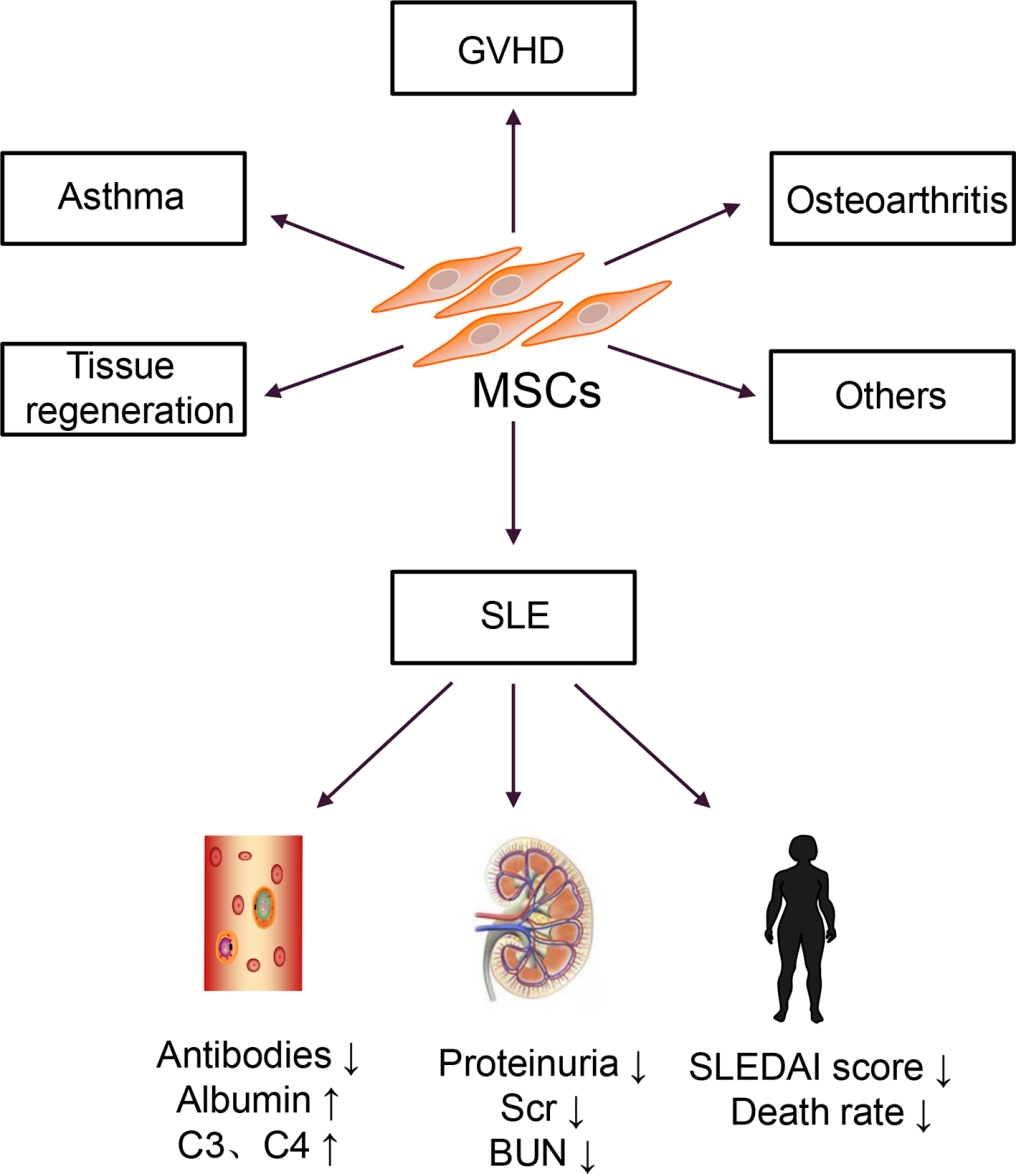
Clinical applications of MSCs.

In the 1960s and 1970s, Friedenstein discovered a cell that could differentiate and adhere to plastic under culture conditions (17). In 1991, Caplan named these cells mesenchymal stem cells (MSCs) (18). Then, bone marrow-derived MSCs (BM-MSCs) were isolated and cultured *in vitro* and transferred back into patients with hematologic malignancies (19). No transplantation-related adverse reactions were observed in this report. Autologous BM-MSCs are more accessible to obtain than allogeneic BM-MSCs, and they do not induce immune rejection; thus, they were used for disease treatment (20). However, a clinical study of autologous MSC transplantation for SLE treatment revealed that autologous MSCs increased Treg cells but had no effect on disease activity and could not reduce the patient’s clinical symptoms (21). Allogeneic MSC transplantation has a more extensive therapeutic range and therapeutic potential than autologous transplantation. This approach is widely used to treat various diseases, including graft versus host disease (GVHD), osteoarthritis, and asthma, and in the regeneration and repair of damaged tissues (22). Therefore, research on MSCs has shifted from basic research to clinical applications, particularly in SLE, as shown in **Figure 1**.

MSCs can also be successfully isolated from the umbilical cord tissues and placenta, and the properties and functions of these MSCs were similar to BM-MSCs; however, compared with BM-MSCs, these cells exhibited lower immunogenicity and more vigorous proliferation and differentiation abilities (23, 24). Subsequently, several allogeneic MSC transplantation for patients with active and refractory SLE were carried out (25–27). A series of results have been obtained that the doses of immunosuppressive drugs used in patients with SLE reduced, and the mortality rate significantly decreased. However, MSC therapy for SLE is currently in the clinical stage. Although most clinical studies have confirmed that MSCs are effective for SLE treatment, many challenges remain to overcome before clinical application.

## Molecular Mechanisms of MSCs in SLE

### MSCs Regulate Adaptive Immune Cells

**Figure 2:**
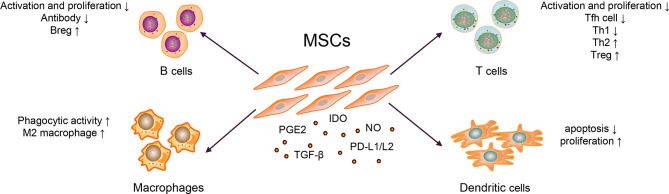
Mechanisms of MSC Therapy in SLE.

The immunosuppressive effect of MSCs is essential for MSC therapy. MSCs can express prostaglandin E2 (PGE2) (28), transforming growth factor-beta (TGF-β) (29), nitric oxide (NO) (30), C–C motif chemokine ligand 2 (CCL2) (31), indoleamine-pyrrole 2,3-dioxygenase (IDO) (32), interleukin-10 (IL-10) (29, 33), and programmed cell death-1 ligands (PD-L1 and PD-L2) (34). Transplanted MSCs can act on tissues or organs through cell–to–cell contact, secrete cytokines and extracellular vesicles (EVs), which further inhibit the production of pro-inflammatory cytokines, and exert immunosuppressive effects, as shown in **Figure 2** (35–37).

Abnormally activated B cells in patients with SLE exert multiple functions, such as producing large quantities of autoantibody (*e.g.*, anti-dsDNA and ANA), secreting pro-inflammatory cytokines (*e.g.*, IL-6 and IFN-γ), and anti-inflammatory cytokines (*e.g.*, IL-10 and TGF-β) (1). MSCs could inhibit B cells differentiation into plasma cells and antibody production *via* soluble factors and cell–to–cell contact involving the PD-1/PD ligand pathway (35, 36). Regulatory B cells (Bregs) exert immunosuppressive functions at least partly through the production of IL-10 and TGF-β in SLE (38, 39). MSCs can induce the expansion of Bregs and inhibit excessive inflammatory responses in a murine lupus model (33). Currently, whether MSCs could affect the expression of B cell co-stimulatory molecules and cytokine production is unknown.

Abnormal activation of T cells, imbalance of Th1/Th2, and other cell subsets are generally involved in the pathogenesis of SLE. The serum IL-17 from patients with SLE were significantly higher than healthy controls, which positively correlated with the SLEDAI score (40). It has been widely reported that T follicular helper (Tfh) cells could help B cells produce autoantibodies and form immune complexes, which caused tissue and organ damage, and eventually aggravated the condition of SLE patients (2). MSCs inhibited the differentiation of naïve CD4^+^ T cells into Tfh cells through cell–to–cell contact and the activation of iNOS, decreased the production of IL-21, alleviated lupus nephritis, and prolonged the survival rate of lupus-prone mice (30, 41). Similarly, Th1/Th2 subgroups in patients with SLE were unbalanced (biased toward Th1) and released pro-inflammatory cytokines; they have been considered important for disease and promote SLE progression (42). Additionally, studies have shown that MSCs could inhibit T cell activation in a dose-dependent manner; inhibit the differentiation of CD4^+^ T cells into Th1, Th17, and Tfh cells; promote Treg proliferation and secretion of IL-10, reduce the ratio of Th1/Th2; and restore the proportion of Treg/Tfh cells, thereby correcting the abnormally activated T cells and cell subsets in patients with SLE (41, 43). In addition, it is reported that after treating thirty refractory SLE patients with human umbilical cord-MSCs for three months, the Treg subgroups and the levels of TGF-β in the peripheral blood were increased (40). In contrast, the expression levels of Th17 cells and IL-17, tumor necrosis factor (TNF-α), and other pro-inflammatory factors were significantly decreased (40). They further co-cultured human umbilical cord-MSCs with peripheral blood mononuclear cells from SLE patients and found that MSCs could upregulate the expression of TGF-β and Treg cells in a dose-dependent manner *in vitro* (40). Researchers also employed a combination of MSCs with five SLE clinical drugs, *viz*., prednisone, dexamethasone, cyclosporin A, mycophenolate mofetil, and rapamycin, in animal experiments. These drugs could improve the therapeutic effect of MSCs, thereby enhancing the functions of Treg and alleviating the drug cytotoxicity (44).

### MSCs Regulate Innate Immune Cells

The innate immune response is the first line of defense against viral invasion *in vivo*. Recent studies have revealed that the innate immune response plays a vital role in SLE progression by initiating and maintaining an adaptive immune response.

Macrophages have two functional states, often exhibited pro- (M1) as well as anti-inflammatory (M2) properties (45). In SLE, macrophages have the defective phagocytic ability and are abnormally activated, promoting disease progression (3). When co-cultured with macrophages, MSCs exerted an immunomodulatory effect by upregulating anti-inflammatory and downregulating pro-inflammatory molecules of macrophages in a murine lupus model (46). Except for regulating macrophage polarization, the study also revealed that MSCs could enhance the phagocytic activity of macrophages, thereby alleviating disease activity in a murine model (46, 47). In other diseases, such as leukemia, MSCs could also help host macrophages to repair the damaged bone marrow microenvironment by reprogramming macrophages (48).

DCs play a critical role in activating T cells and B cells (5, 49). Two types of DC subsets have been identified in *Homo sapiens*: myeloid DCs and plasmacytoid DCs (50). In the pathogenesis of SLE, plasmacytoid DCs were considered the primary source of type I interferon (IFN), which promoted the activation of T and B cells (51). Similarly, co-stimulatory molecules overexpressed by myeloid DCs could accelerate T cell maturation, promote T cells differentiation into pro-inflammatory cells and lead to organ damage *in vitro* (5). It confirmed that MSCs could inhibit the maturation and function of DCs and reduced the expression of presentation molecules, such as human leukocyte antigen-DR and co-stimulatory molecules, such as CD80 and CD86 (52). MSCs could also induce the production of regulatory DC (DCregs) and escaped apoptosis, further enhancing phagocytosis’s ability and inhibiting T cells’ activation and proliferation (53, 54). In addition, MSCs inhibited the secretion of TNF-α by DCs and upregulated the secretion of IL-10 (55). But these mechanisms are known little in SLE. As DCs is essential for the pathogenesis of SLE, these functions of MSCs need to be confirmed in murine model or patients with SLE. Later, it was revealed that the numbers of tolerogenic CD1c^+^ DCs in the peripheral blood and the levels of serum FLT3L in patients with SLE significantly decreased (56). After transplanting of umbilical cord-MSCs, the significantly upregulated levels of FLT3L promoted the proliferation and inhibited the apoptosis of tolerogenic CD1c^+^ DCs, thereby improving the condition of lupus (56). In SLE, the activity of myeloid-derived suppressor cells impaired in both murine and humans (57) could promote Th17 cells and Treg differentiation and shift the ratio of Th17/Treg, thereby promoting the progression of SLE (58). MSC infusion could restore the activity of myeloid-derived suppressor cells in inflammatory and autoimmune diseases, such as Sjögren syndrome (28, 59); however, it remains unclear in SLE.

## Current Status and Challenges of the Application of MSCs in SLE

There are currently thirteen clinical studies on the treatment of SLE with MSCs registered at www.clinicaltrials.gov, among which nine have completed, and four are in progress. Many studies suggested that MSC transplantation could effectively improve the clinical symptoms of active and refractory SLE patients; reduce the SLEDAI score; decrease the levels of proteinuria, autoantibodies, and complements; and reverse multiple organ damage (26, 60).

A meta-analysis on MSC therapy in a murine lupus nephritis model was conducted from October 2009 to October 2020 and revealed that MSC therapy increased the levels of serum albumin and reduced the levels of dsDNA and proteinuria (61). Moreover, MSCs could reduce the levels of IL-2, IL-12, IL-17, IFN-γ and improve the renal sclerosis score (61). Also, allogeneic MSC transplantation was used for fifteen patients with active and refractory SLE and followed up for 17.2 ± 9.5 months (25). No severe toxicities or adverse events were reported (25). All patients attained disease remission, and the SLEDAI score, anti-dsDNA levels, and 24h proteinuria levels markedly decreased within one year. One year later, two patients experienced a relapse of proteinuria (25). This approach was a good starting point for MSC therapy of SLE. Subsequently, a six-year follow-up observation of allogeneic MSC transplantation for refractory SLE found that all patients tolerated the treatment well, with no increase in the risk of tumor formation or infection (27). Furthermore, MSC therapy decreased the SLEDAI score, the levels of autoantibodies, proteinuria and increased serum albumin levels (62). The latest long-term retrospective study reported that MSC transplantation-related mortality was only 0.2%, confirming the effectiveness and safety of MSC transplantation (63). To date, MSC therapy was the most promising treatment for SLE, particularly for patients who do not respond well to traditional therapies.

Although most studies indicated that MSC transplantation could improve the disease condition of patients with SLE, a few have shown that MSC therapy was ineffective. It was worth noting that no severe adverse effects were observed after autologous BM-MSC transplantation. Although BM-MSCs increased the numbers of CD4^+^CD25^+^FoxP3^+^ cells, they did not improve disease conditions, even in young patients (21, 64). Murine BM-MSCs inhibited the deposition of immune complex in the glomerulus and restrained lymphocytic infiltration and glomerular proliferation in lupus animal models (36). However, they did not affect the production of anti-dsDNA or proteinuria (36). In addition, based on standard immunosuppressive therapy, twenty-five patients with SLE were recruited to treat with human umbilical cord MSCs (dose 2 × 10^8^ cells/person) in 2012 (NCT01539902) (65). However, the clinical study was terminated after treating eighteen patients and revealed that the levels of proteinuria, serum albumin, complement, SLEDAI score, and renal function in the MSC therapy group had no significant difference compared to the placebo group (65). However, the authors did not provide specific reasons for this failure. Hence, the therapeutic effects of MSCs must be confirmed in large-scale clinical studies.

Notably, intravenous infusion of MSCs is complicated and may have serious side effects, such as vascular occlusion and the induction of tumor formation. Considering the safety of MSC transplantation, many investigators have focused on EVs derived from MSCs. Compared with MSCs, EVs derived from MSCs exhibited similar immunosuppressive functions in several studies *in vitro* as cell-free therapy and showed high safety (66, 67). However, there is currently no standard and high-efficiency extraction method for EVs, which leads to low production yields and increased heterogeneity of MSC EVs (68). Notably, EVs derived from MSCs have not been used in clinical studies of SLE.

## Potential Causes of MSC Therapy Inefficacy in SLE Treatment

In the past few decades, there have been successes and failures in using MSCs to treat SLE. There is no evidence that MSCs are unsafe or promote the progression of SLE. However, reports showed that MSCs could secrete cytokines with strong pro-inflammatory effects, such as IL-6 (69, 70), which might be related to some controversy in their application. Also, MSCs are susceptible to aging due to the influence of the surrounding environment (71). The etiology and pathogenesis of SLE are still unclear. The microenvironment of patients with SLE is complicated, causing the therapeutic effects of intravenously infused MSCs can be influenced by many factors. We summarize the potential causes of MSC therapy inefficacy in SLE from the following aspects: the defective BM-MSCs in patients with SLE, the expansion of MSCs *in vitro*, and the complex microenvironment in patients with SLE. It is worth noting that studies in many other diseases have confirmed that most intravenously infused MSCs could be trapped and cleared in the lung (72–74), which may be one of the reasons for MSC therapy inefficacy.

### Defective BM-MSCs in Patients With SLE

SLE is an autoimmune disease that is genetically inherited, with patients showing disease-related susceptibility genes (75, 76). It is well known that long-term use of high doses of immunosuppressive agents could increase the risk of bone marrow suppression in patients, which aggravated complications such as infection and anemia. MSCs from the bone marrow of patients with SLE were defective (14). Hence, SLE is considered a type of stem cell-mediated disease, resulting in weakened HSCs growth and differentiation (14, 77). The morphology of BM-MSCs in patients with SLE was similar to that of healthy controls, and both exhibited the typical immunophenotype, positive for CD44, CD73, and CD105, and negative for CD34, CD19, CD45, and other hematopoietic cell indicators; however, BM-MSCs from patients with SLE exhibited proliferation, differentiation, migration, and homing ability defects and are more prone to senescence and apoptosis (14). Moreover, their ability to secrete cytokines is weakened, causing a decrease in the inhibitory effect on T and B cells and other immune cells, thereby promoting the progression of SLE (71, 77). In addition, BM-MSCs are affected by age. As donors grow older, BM-MSCs tend to senescence, and their functions gradually weaken, resulting in poor effects after transplantation (77).

If combined transplantation of MSCs and HSCs, MSCs could promote the transplantation of HSCs, enhance hematopoietic function, and improved GVDH condition *in vivo* (78). This finding indicates that MSCs have potent roles in promoting body repair while exerting immunosuppressive effects. However, it is unknown whether MSCs transplanted into the body could promote the recovery of BM-MSC function in SLE. Additional clinical data are required to confirm these findings. If appropriate methods are used to modify the BM-MSCs of patients with SLE *in vitro* and then re-inject them into the body, the function of autologous defective MSCs in these patients is expected to be restored.

### Effect of *In Vitro* Expansion of MSCs

A murine model showed when the generation of MSCs expanded *in vitro* is low, the cells possess a stronger ability to home to damaged tissues (79). However, the morphology and function of younger MSCs are unstable, resulting in unknown effects. When the number of MSCs is lower, fewer cells can be used for transplantation. And a low number of MSCs could promote lymphocyte proliferation while a larger dose always has an inhibitory effect on lymphocyte proliferation (80). Therefore, to achieve the best therapeutic effect, MSCs must be cultured *in vitro* to obtain sufficient cells.

However, there is currently no standardized system for the isolation, culture, and expansion of MSCs. When MSCs are cultured and expanded *in vitro*, gene mutations may occur because of the culture system and conditions used, resulting in expansion-related senescence, weakened proliferation and differentiation ability, reduced adhesion, and homing ability (81). Also, if MSCs are expanded *in vitro* for a long time, they could gradually lose the ability to recognize endogenous tissues and exhibit weakened genetic stability (82); therefore, when these cells are transplanted into the body, their therapeutic effect may decrease, or they may pose a safety risk.

### Complex Microenvironment in Patients With SLE

MSCs were mostly trapped in the lungs when injected intravenously into the body (83) and could not be detected after 7–14 days [65]. However, due to the different microenvironments of patients with SLE, the residence time and efficacy of MSCs differ; when harmful factors damaged local tissues, the residence time of MSCs *in vivo* could be extended, promoting the repair of damaged tissues (84). In addition, high-level inflammatory factors could enhance the immunosuppressive effect of MSCs on immune cells by simulating the inflammatory microenvironment of patients *in vitro* (85).

Evidence showed an imbalance in Th1/Th2 and other cell subpopulations in patients with SLE, which significantly increased the levels of the pro-inflammatory cytokines IL-6, TNF-α, and IL-1β (86). IL-6 and IL-1β were known to drive Th17 differentiation and promote the levels of serum IL-21 and IL-17 which correlated with disease activity (87, 88). When MSCs exposed to IL-6, the stemness of MSCs was enhanced through an ERK1/2-dependent mechanism (89); however, whether IL-6 could reduce the immunosuppressive function of MSCs still unclear. Therefore, it needs to further investigate the specific effect of IL-6 on MSCs. In patients with SLE, high concentrations of serum TNF-α could significantly inhibit the migration and homing capacity of SLE BM-MSCs *via* TNF receptor I (90). Also, anti-TNF therapies for rheumatic diseases led to the formation of anti-dsDNA and drug-induced lupus (91). In addition, the upregulation of renal TNF-α was considered to play a vital role in the activation of local inflammation and formation of tissue damage (92); however, in collagen-induced arthritis, when TNF-α was present in large quantities, increasing the number of MSCs does not relieve the clinical symptoms (93). When co-stimulated with TNF-α and IL-1β, MSCs exhibited pro-inflammatory effects and promoted T cell proliferation and differentiation (94). In summary, the inflammatory environment may induce MSCs to exert pro-inflammatory effects, leading to the failure of MSC therapy in autoimmune diseases, including SLE.

MSCs can secrete anti-inflammatory cytokines such as indoleamine-pyrrole 2,3-dioxygenase and prostaglandin E2, as well as pro-inflammatory cytokines such as IL-6 and TNF-α (69), which may accelerate disease progression. Another study revealed that IL-6 silencing could weaken the inhibition of the proliferation of activated T cells (95). Therefore, MSCs may have dual effects on the disease.

## Novel Mechanisms and Directions of MSCs in SLE Treatment

MSCs have strong immunomodulatory plasticity and could be easily influenced by the microenvironment, which is among the reasons why MSC therapy is ineffective. Thus, MSC modifications, such as genetic and preconditioning modifications, could avoid the influence of the environment. The former alters MSCs by inserting a gene, whereas the latter alters MSCs using chemical and/or physical factors *in vivo*, thereby overexpressing specific genes and improving the efficacy of disease treatment. Modified MSCs are now widely used to treat tumors, cardiovascular diseases, neurological diseases, bone, and joint diseases, and so on (96).

Pro-inflammatory cytokines contribute to the pathogenesis of SLE; however, for MSCs, strong inflammatory cytokines were effective attractors that could activate the immune-suppressive function, whereas low levels of inflammatory factors could reduce the immune-suppressive role or even trigger the immune system (97, 98). In a murine model, transplanted IL-37 overexpressed MSCs inhibited the inflammatory microenvironment *in vivo*, prolonged survival, and reduced SLE-like symptoms (99). Similarly, pretreated MSCs with media containing pro- or anti-inflammatory cytokines or related molecules such as poly (I:C) and glucocorticoids could enhance the immunosuppression of MSCs (100–102). Pretreated MSCs with IFN-γ could increase IDO (32) and significantly inhibited splenic B cells’ proliferation and the production of antibodies (103). The pretreatment of MSCs with IL-1β significantly increased the number of Treg and Th2 cells and decreased Th1 and Th17 cells (104). Besides, if IFN-γ co-cultured with any of the three other pro-inflammatory cytokines, *viz*., TNF-α, IL-1α, and IL-1β, the adhesion, migration, and homing abilities of MSCs could be enhanced (105). Modified MSCs with IL-10 could inhibit tumor growth by reducing the production of IL-6 (106). These results indicate that MSC modification could enhance the immunosuppression of MSCs, providing a new and feasible direction for SLE therapy.

The aging phenotype of MSCs could be wholly or partially reversed by inhibiting MSC senescence-related genes, which improves the immune regulation function *in vitro* (107, 108). Recently, several studies demonstrated that the pretreatment of MSCs with rapamycin and Dickkopf-1 reversed the senescence of MSCs and improved the immune regulation of MSCs (108–110). There are also other ways to modify MSCs and reverse senescence phenotype, such as pretreated MSCs with hypoxia or by upregulating the expression of CBX4 or Erb-B2 receptor tyrosine kinase 4 (ERBB4), which could change the senescence phenotype, reduce the expression of senescence-associated β-gal, and maintain the stemness of MSCs (107, 111–113). In contrast, upregulated CD146, CD264, SIRT3, and TLR3 expression levels in MSCs increase senescence (114–117). MSC senescence is unavoidable in SLE treatment. If the senescence phenotype of MSCs is modified by various methods, the function of MSCs can be improved, thereby enhancing their therapeutic ability.

Several studies have revealed that increased the expression of homing molecules and cell surface receptors, such as CC chemokine receptors 1 (CCR1) (118), C–C motif chemokine ligand 2 (CCL2) (31), C–X–C motif chemokine receptors 2 (CXCR2) (119), CXCR4 (120) by modifying MSCs could promote the therapeutics of MSCs.

However, it is controversial whether the migration and homing of MSCs to damaged tissues are required for their immunomodulation effects. For the local immune response of MSCs, the therapeutics of MSCs may be associated with its migration and homing abilities. It has been reported that the overexpression of CCR1, CXCR2, and CXCR4 in MSCs or modified MSCs with biomimetic extracellular matrices and poly (dimethylsiloxane) could stimulate more MSCs to migrate to the lesion sites, secrete more anti-inflammatory cytokines, and accelerate tissue healing (118–122). The pretreatment of MSCs with miR-9-5p or TNF-α could also improve the migration ability of MSCs, whereas the inhibition of miR-9-5p reduced MSC migration (123, 124). For the systemic immune responses of MSCs, many studies observed the phenomenon that most of the MSCs were trapped in the lung after IV infusion in murine models (72–74). For the mechanisms, the MSCs trapped in the lung after IV infusion may secret bioactive molecules and EVs into the blood and efficiently regulate systemic immune responses (125, 126). For example, MSCs trapped in the lung with higher expression of the gene for a multifunctional anti-inflammatory protein tumor necrosis factor-α stimulated gene/protein 6 (TSG-6) could efficiently regulate systemic immune responses in lung injury mice (125). Meanwhile, the overexpression of CCL2 in MSCs from patients with SLE could improve MSC immunoregulatory abilities both *in vitro* and *in vivo*, whereas the knockdown of CCL2 from normal MSCs led to a weakened immunoregulatory power (31). This may also correlate to the bioactive molecules secreted by MSCs. However, the specific molecular mechanisms of these bioactive molecules and EVs regulating the systemic immune responses need further studies.

Studies showed the immunomodulatory of MSCs is partly the result of EVs, which play an increasingly important role in MSC therapy (127, 128). As a novel cell-free therapy, EVs could deliver specific molecules to target tissues or organs and exhibit nearly the same immunomodulation ability as MSCs (128, 129). EVs derived from modified MSCs are widely used in several diseases *in vitro* (37, 127–129). Exosomes derived from miR-122-modified MSCs could improve the sensitivity of tumor cells to drugs and increase the effect of drugs on cancer treatment (127). In the rat models of spinal cord injury, exosomes derived from miR-126-modified MSCs could promote the recovery of injury volume and trigger the regeneration of axons (128). Moreover, exosomes from Akt-modified MSCs in the acute myocardial infarction rat models could reduce myocardial cell apoptosis, increase cardiac regeneration, and improve cardiac function (129). In rheumatoid arthritis (RA) murine model, exosomes derived from miR-150-5p modified MSCs could decrease the regeneration of synoviocytes and reduce joint destruction, thereby being the potential treatment for RA (37). However, EVs derived from MSCs remains limited in murine models and patients with SLE.

## Future Perspectives

MSCs are currently used to treat patients with active and refractory SLE, and a series of promising results have been obtained. However, MSCs do not always exhibit strong immunosuppressive function and may lose their therapeutic effect under the influence of many factors. This may occur for the following reasons: defects of BM-MSCs in patients with SLE, the impact of MSCs culture *in vitro*, and the complex microenvironment of patients with SLE. To maximize the therapeutic effects of MSCs or EVs derived from MSCs *in vivo*, MSCs need to be pretreated by various means, including pro- and anti-inflammatory cytokines, improving their senescence and enhancing their migration and homing ability.

However, MSC modification must also be confirmed to determine whether MSC gene mutation will occur or if the transplantation of MSCs will harm the body in the long term, including serious problems such as tumorigenesis and teratogenesis. No studies have focused on whether the modification of MSCs alters their safety. In the long term, MSC modification may improve the therapeutic effects of MSCs in autoimmune diseases, particularly in SLE.

MSCs are in the early stages of clinical application and are typically combined with hormonotherapy. Whether hormone therapy can be discontinued using modified MSCs should be examined. In addition, MSC therapy improves but does not completely cure SLE. Thus, whether modified MSCs can cure SLE requires further analysis. Limited by their high cost, safety concerns, and lower SLEDAI scores of disease conditions, MSCs are rarely used in patients with mild SLE. However, comprehensive studies of MSCs and improvements in their preparation process can reduce costs and significantly expand the application of MSCs. MSCs may be more effective in patients with mild SLE or preempted for those with a genetic background of SLE, which may relieve these patients’ conditions.

## Author Contributions

AL, FG and QRP wrote the manuscript and designed the figures. SC, JC, and H-FL revised the manuscript. All authors contributed to the article and approved the submitted version.

## Funding

This study was supported by National Natural Science Foundation of China (no.82070757), the Project of “Dengfeng Plan” and Department of established positions for the Zhujiang Scholar from Guangdong Medical University, and Guangdong Basic and Applied Basic Research Foundation (no.2019A1515012203), the Zhanjiang City Program for Tackling Key Problems in Science and Technology (no. 2019B01179).

## Conflict of Interest

The authors declare that the research was conducted in the absence of any commercial or financial relationships that could be construed as a potential conflict of interest.

## Publisher’s Note

All claims expressed in this article are solely those of the authors and do not necessarily represent those of their affiliated organizations, or those of the publisher, the editors and the reviewers. Any product that may be evaluated in this article, or claim that may be made by its manufacturer, is not guaranteed or endorsed by the publisher.
